# Improved Functional Expression of Cytochrome P450s in *Saccharomyces cerevisiae* Through Screening a cDNA Library From *Arabidopsis thaliana*

**DOI:** 10.3389/fbioe.2021.764851

**Published:** 2021-12-09

**Authors:** Lihong Jiang, Chang Dong, Tengfei Liu, Yi Shi, Handing Wang, Zeng Tao, Yan Liang, Jiazhang Lian

**Affiliations:** ^1^ Key Laboratory of Biomass Chemical Engineering of Ministry of Education, College of Chemical and Biological Engineering, Zhejiang University, Hangzhou, China; ^2^ Hangzhou Global Scientific and Technological Innovation Center, Zhejiang University, Hangzhou, China; ^3^ Ministry of Agriculture Key Laboratory of Molecular Biology of Crop Pathogens and Insects, Institute of Biotechnology, Zhejiang University, Hangzhou, China

**Keywords:** cytochrome P450 enzymes, *Arabidopsis thaliana* cDNA overexpression library, biosensor, betaxanthin, Z-α-santalol, *Saccharomyces cerevisiae*

## Abstract

Cytochrome P450 enzymes (P450s) are a superfamily of heme-thiolate proteins widely existing in various organisms and play a key role in the metabolic network and secondary metabolism. However, the low expression levels and activities have become the biggest challenge for P450s studies. To improve the functional expression of P450s in *Saccharomyces cerevisiae*, an *Arabidopsis thaliana* cDNA library was expressed in the betaxanthin-producing yeast strain, which functioned as a biosensor for high throughput screening. Three new target genes *AtGRP7*, *AtMSBP1*, and *AtCOL4* were identified to improve the functional expression of *CYP76AD1* in yeast, with accordingly the accumulation of betaxanthin increased for 1.32-, 1.86-, and 1.10-fold, respectively. In addition, these three targets worked synergistically/additively to improve the production of betaxanthin, representing a total of 2.36-fold improvement when compared with the parent strain. More importantly, these genes were also determined to effectively increase the activity of another P450 enzyme (CYP736A167), catalyzing the hydroxylation of *α*-santalene to produce Z-α-santalol. Simultaneous overexpression of *AtGRP7*, *AtMSBP1*, and *AtCOL4* increased *α*-santalene to Z-α-santalol conversion rate for more than 2.97-fold. The present study reported a novel strategy to improve the functional expression of P450s in *S. cerevisiae* and promises the construction of platform yeast strains for the production of natural products.

## Introduction

Cytochrome P450 enzymes (P450s), first discovered in the early 1960s, are a superfamily of heme-thiolate proteins widely existing in animals, plants, and microorganisms (Elfaki et al., 2018; Guengerich et al., 2016). P450s play important roles in the metabolic networks, due to various biocatalytic activities such as oxidation, epoxidation, hydroxylation, and demethylation (Stavropoulou et al., 2018). More importantly, P450s are involved in the biosynthesis of many natural products, such as opioids (Galanie et al., 2015), artemisinic acid (Paddon et al., 2013), and glycyrrhetinic acid (Zhu et al., 2018). As a thoroughly studied model organism, *Saccharomyces cerevisiae* has advantages of clear genetic background, easy cultivation, and post-translational processing capability (Schuler and Werck-Reichhart 2003). Most importantly, the inner membrane systems of *S. cerevisiae* allow the functional anchoring of P450s and cytochrome P450 reductases (CPRs) (Hausjell et al., 2018). Therefore, *S. cerevisiae* is often selected as a preferred host for functional expression of P450s and accordingly biosynthesis of natural products.

Although a variety of P450s have been successfully expressed in *S. cerevisiae*, low expression level and activity have become the biggest challenge for fundamental and biotechnological application studies of P450s (Jiang et al., 2021). Accordingly, different strategies have been implemented to increase the expression level and/or activity of P450s in *S. cerevisiae*, including N-terminal truncation, protein molecular modification of P450s through protein engineering, and co-expression with CPRs (Jiang et al., 2021). Unfortunately, the effects of these engineering strategies are often varied case by case. In other words, it is of great demand to develop a generally applicable strategy to improve the functional expression of a wide range of P450s in *S. cerevisiae*.

In plants, there are many kinds of P450s, interacting with a variety of substrates to participate in the synthesis and degradation of alkaloids, terpenes, flavonoids, fatty acids, plant hormones, and signal molecules (Bolwell et al., 1994). Thus, plants have evolved a complex gene regulation system to control the expression and folding of P450s (Yang et al., 2016). It is speculated that the introduction of key genes involved in the plant regulatory network may have positive effects on the functional expression of a series of P450s in yeast.

**FIGURE 1:**
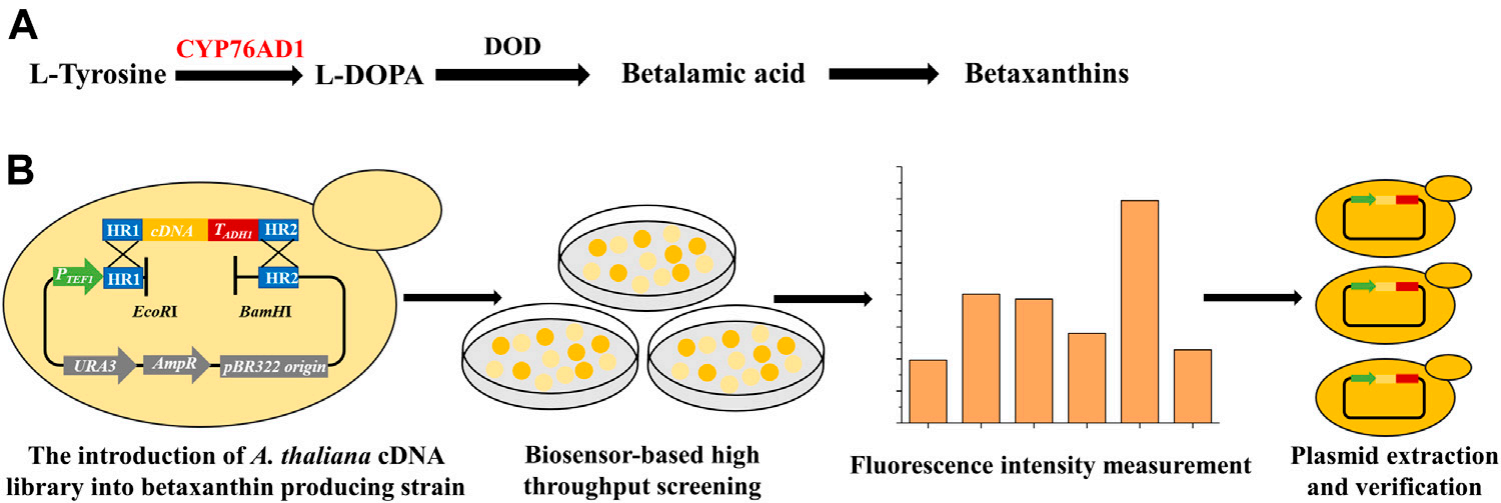
Establishment of a high throughput screening method for identifying new target genes of the *A. thaliana* cDNA library to improve the functional expression of P450s. **(A)** The betaxanthin biosynthetic pathway starting from L-tyrosine, including a P450 enzyme (CYP76AD1, shown in red) and an L-DOPA dioxygenase (DOD). CYP76AD1 has been determined to be rate-limiting for betaxanthin production, indicating that the production of betaxanthin (yellow color or fluorescence) can function as a biosensor for the expression level of *CYP76AD1*. **(B)** Workflow of the genome-scale engineering experiments. The *A. thaliana* cDNA overexpression library was introduced into the betaxanthin-producing yeast (yJS1256, biosensor strain) *via in vivo* homologous recombination. Clones with obvious color changes were selected and inoculated into SCD-URA medium in 96 deep-well plates and the fluorescence intensities were measured using a microplate reader. Then the isolated plasmids were re-transformed into the biosensor strain and the corresponding genes were identified by DNA sequencing.

The present study aims to identify key genes that can improve the functional expression of P450s in *S. cerevisiae* through screening a genome-scale cDNA library from *Arabidopsis thaliana*, a model plant widely used in plant genetic, cellular, developmental, and molecular biology researches (Hayashi and Nishimura 2006). Firstly, an *A. thaliana* cDNA library was overexpressed in *S. cerevisiae*, which was combined with biosensor-based high-throughput screening (Figure 1A) to identify target genes that can improve the functional expression of a P450 (*CYP76AD1*) in yeast. Then, the synergistic interactions and molecular mechanisms of these newly identified targets were further explored. Finally, these plant genes were overexpressed to increase the activity of another P450 protein (encoded by *CYP736A167*) involved in the hydroxylation of *α*-santalene to Z-α-santalol. The present study promises the establishment of a platform yeast strain for functional expression of a wide variety of P450s.

## Materials and Methods

### Strains and Plasmids

The yeast biosensor strain yJS1256 for high throughput screening was kindly provided by Prof. Dueber from the University of California at Berkeley (DeLoache et al., 2015). The *A. thaliana* cDNA library under the control of *ADH1* promoter and *ADH1* terminator in plasmid pGADT7-AD was kindly provided by Prof. Zeng Tao from Zhejiang University. To facilitate the construction of the *A. thaliana* cDNA overexpression library in *S. cerevisiae*, a helper plasmid pRS416-TEF1p was constructed by cloning *TEF1* promoter, upstream and downstream homology arms of the cDNA library, as well as *EcoR*I and *BamH*I restriction sites into pRS416. Then pRS416-TEF1p was digested by *EcoR*I and *BamH*I and co-transformed with the PCR amplified cDNA library fragments with homology arms into yJS1256 for *in vivo* assembly (Figure 1B). The identified plasmids from the *A. thaliana* cDNA library were isolated using a ZymoPrep Yeast Plasmid Miniprep Kit (Zymo Research, Irvine, CA) and transformed into *Escherichia coli* DH5α for plasmid amplification. The Z-α-santalol producing *S. cerevisiae* strain was constructed by integrating four copies of the *α*-santalene synthase (SAS) gene (Dong et al., 2020) and one copy of *CYP736A167* and *CPR2* from *Santalum album* (XII5 locus) into the genome of BY4741 (*MAT*a *his△1 leu2△0 met15△0 ura3△0*), *via* CRISPR-Cas9-mediated genome editing technology (Lian et al., 2017; Lian et al., 2019). In addition, the mevalonate (MVA) pathway genes were overexpressed to enhance the precursor supply for Z-α-santalol biosynthesis, with *tHMG1-ERG8*-*ERG13*-*ERG20*-*ERG12* overexpression cassettes and *EGR10*-*MVD1*-*IDI1*-*tHMG1* overexpression cassettes integrated into X4 and XI3 loci, respectively. Q5 polymerase, T4 DNA ligase, and all restriction enzymes used were purchased from New England Biolabs (Ipswich, MA). All chemicals were bought from Sigma (Sigma Aldrich, St. Louis, MO) unless otherwise stated.

### Growth Conditions

*E. coli* strain DH5α for cloning and plasmid propagation was cultured at 37°C in Luria-broth (LB) medium containing 100 μg/ml ampicillin. Yeast strains were cultivated in standard yeast peptone dextrose (YPD) medium consisting of 2% glucose, 2% peptone, and 1% yeast extract. Recombinant yeast strains were grown on complete synthetic (SCD-URA) medium consisting of 0.17% yeast nitrogen base (YNB, Difco, Boom, Netherlands), 0.5% ammonium sulfate, and the appropriate amino acid drop-out mix (CSM-URA, MP Biomedicals, Solon, Ohio) supplemented with 2% glucose at 30°C.

### High-Throughput Screening and Fluorescence Intensity Measurement

The *A. thaliana* cDNA overexpression library was constructed by the *in vivo* DNA assembly method in *S. cerevisiae.* After transformation, 10^6^ independent clones were observed on SCD-URA agar plates, indicating at least a 50-fold coverage of the *A. thaliana* cDNA library. For biosensor-based high-throughput screening, 76 clones with the highest yellow color intensities were selected from SCD-URA agar plates and inoculated into 1 ml SCD-URA medium in a 96 deep-well plate. The yeast strains were pre-cultured for 2 days and then inoculated into fresh SCD-URA medium with an initial OD_600_ of 0.1. Mid-log phase yeast cells were collected and diluted 2-fold in ddH_2_O for measuring betaxanthin fluorescence intensity at 498–533 nm using a Tecan microplate reader. The fluorescence intensity (relative fluorescence units; RFU) was normalized to cell density that was determined by the same microplate reader.

### Z-α-Santalol Production and Quantification

Z-α-Santalol producing strains were pre-cultured in SCD-URA medium for 2 days, inoculated into 50 ml fresh medium in 250 ml shaker flasks with an initial OD_600_ of 0.1, and cultured at 30°C and 250 rpm for 5 days. Then 2 ml yeast cells were collected by centrifuge at 12,000×*g* for 2 min and resuspended in 700 μL ethyl acetate to be disrupted by bead milling. The cell lysate was centrifuged for 10 min and the supernatant was filtered for GCMS (SHIMADZU, Japan) analysis on a DB-5MS column. 2 μL of each sample was injected with a 20:1 split mode at 280°C. The initial column temperature was 40°C and kept for 3 min. Subsequently, the temperature was increased to 130°C at a rate of 10°C/min, followed by to 180°C at a rate of 2°C/min and to 300°C at a rate of 50°C/min, and finally kept at 300°C for 10 min. The production of *α*-santalene and Z-α-santalol was quantified using the standard curve method. The *α*-santalene to Z-α-santalol conversion rate was calculated as [Z-α-santalol]/([α-santalene]+[Z-α-santalol]).

### Transcriptomic Analysis

Yeast cells in biological triplicates were grown at 30°C overnight in 5 ml of SCD medium for 2 days and inoculated into 50 ml SCD medium in 250 ml shaker flasks with an initial OD_600_ of 0.1. Then cells were harvested in the early stationary phase by centrifugation at 4,000 g for 15 min at 4°C, with the total RNA extracted and sequenced (RNA-Seq) by Shanghai Majorbio Bio-pharm Technology Co., Ltd. RNA-Seq data manipulation and differential gene expression profiling were performed on the free online Majorbio Cloud Platform (www.majorbio.com) with default settings. The RNA-Seq data are available from the NCBI Sequence Read Archive, with an accession number PRJNA760804.

## Results

### Biosensor-Based High Throughput Screening of *A. thaliana* cDNA Overexpression Library in *S. cerevisiae*

A major challenge for P450 engineering is the lack of a high throughput screening method. The betaxanthin-producing yeast strain yJS1256 developed by the Dueber group (DeLoache et al., 2015) was employed as a biosensor for high-throughput screening of yeast strains with improved functional expression of P450s*.* The biosensor is composed of a P450 enzyme mutant (CYP76AD1^W13L−F309L^) from *Beta vulgaris* and an L-DOPA (dihydroxyphenylalanine) dioxygenase (DOD) from *Mirabilis jalapa*, which can catalyze the conversion of l-tyrosine to produce betaxanthin (Figure 1A). In addition, two transporter genes, encoding Qdr2p and Yor1p, were deleted to accumulate betaxanthin intracellularly (Savitskaya et al., 2019). With the introduction of an additional copy of CYP76AD1^W13L−F309L^, the production of betaxanthin was significantly improved, indicating that betaxanthin biosynthesis in yeast was limited by CYP76AD1 (Supplementary Figure S1), which was consistent with the previous report (DeLoache et al., 2015). Considering the color and fluorescence characteristics of betaxanthin and rate-limiting of CYP76AD1 in betaxanthin biosynthesis, a biosensor-based high throughput screening method was established to isolate yeast mutants with improved production of betaxanthin (Figure 1B). Then the *A. thaliana* cDNA library was cloned into the single-copy plasmid pRS416 and overexpressed in *S. cerevisiae*. After yeast transformation, 20 clones were randomly selected for diagnostic PCR verification to investigate the diversity of the *A. thaliana* cDNA library in *S. cerevisiae* (Supplementary Figure S2A). Clones with obvious color changes were selected and inoculated into SCD-URA medium to measure the change in fluorescence intensities (Supplementary Figure S2B). Compared with the control strain containing empty plasmid, the fluorescence intensities of 25 clones were increased to varying degrees. The plasmids from these clones were extracted and sequenced to identify the candidate genes which could improve the production of betaxanthin in yeast.

### Verification of the Candidate Genes for Improved Functional Expression of *CYP76AD1*

**FIGURE 2:**
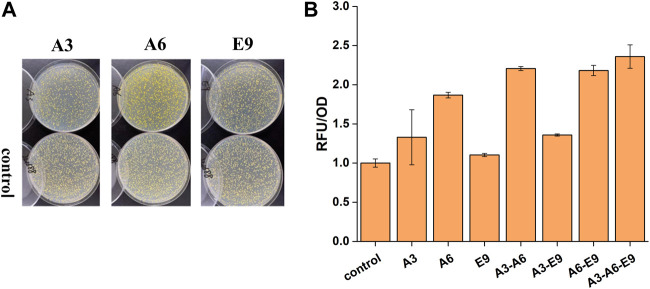
The effects of *AtGRP7* (A3), *AtMSBP1* (A6), and *AtCOL4* (E9) on the functional expression of *CYP76AD1* and accordingly the production of betaxanthin. **(A)** The change of clone color on agar plates after transformation of the target genes. **(B)** The change of fluorescence intensity after transformation of the target genes, as well as synergistic/additive interactions between *AtGRP7* (A3), *AtMSBP1* (A6), and *AtCOL4* (E9). Error bars represented the mean ± s.d. of biological triplicates. RFU/OD refers to the fluorescence intensity normalized to cell density.

After the extraction of plasmids from the isolated clones, they were re-transformed into the biosensor strain yJS1256 to verify the ability of these genes to improve the functional expression of *CYP76AD1* and accordingly the production of betaxanthin in yeast. Three plasmids, A3, A6, and E9 harboring the genes of *A. thaliana* glycine-rich RNA-binding protein (*AtGRP7*), membrane steroid binding protein 1 (*AtMSBP1*), and *A. thaliana* CO-like four protein (*AtCOL4*), showed 1.32-, 1.86-, and 1.10-fold improvement in betaxanthin production, respectively (Figures 2A,B). AtGRP7 (A3) participates in the negative feedback loop of circadian rhythm regulation and pre-mRNA splicing and plays an important role in a complex network of transcripts in *A. thaliana* (Koster et al., 2014; Streitner et al., 2010). AtMSBP1 (A6), an ER-located protein, demonstrates steroid-binding activity *in vitro* and is involved in the inhibition of elongation and brassinosteroid signaling (Shi et al., 2011). More importantly, MSBP1 and its homologue MSBP2 can form homomers and heteromers on the ER membrane, which interact with three monolignin P450 enzymes to form MSBP-P450 protein complexes, thereby improving the stability and activity of P450s as well as the production of lignin (Gou et al., 2018). AtCOL4 (E9), a putative novel transcription factor, has transcriptional activation activity and is an important regulator of plant tolerance to abiotic stress (Min et al., 2015).

Afterwards, the synergistic or additive interactions among *AtGRP7*, *AtMSBP1*, and *AtCOL4* were further investigated. Considering the stability of heterologous genes, these gene expression cassettes were integrated into XII5, X4, and XI3 loci of the yeast genome for combinatorial optimization (Figure 2B). When *AtGPR7* (A3) or *AtCOL4* (E9) was expressed together with *MSBP1* (A6), synergistic and/or additive effects on the production of betaxanthin were observed, representing 2.21- and 2.18-fold higher than the control strain, respectively. In contrast, the effect of simultaneous expression of *AtGPR7* (A3) and *AtCOL4* (E9) was not obvious. The highest production of betaxanthin was achieved in the strain with these three genes (A3-A6-E9) being expressed simultaneously, representing a 2.36-fold improvement over the control strain. These results indicated that there might be synergistic interactions between *AtMSBP1* and *AtGRP7*, as well as *AtMSBP1* and *AtCOL4*, but not *AtGRP7* and *AtCOL4*.

### Effects of the *A. thaliana* Target Gene Overexpression on the Synthesis of Z-α-Santalol in *S. cerevisiae*

**FIGURE 3:**
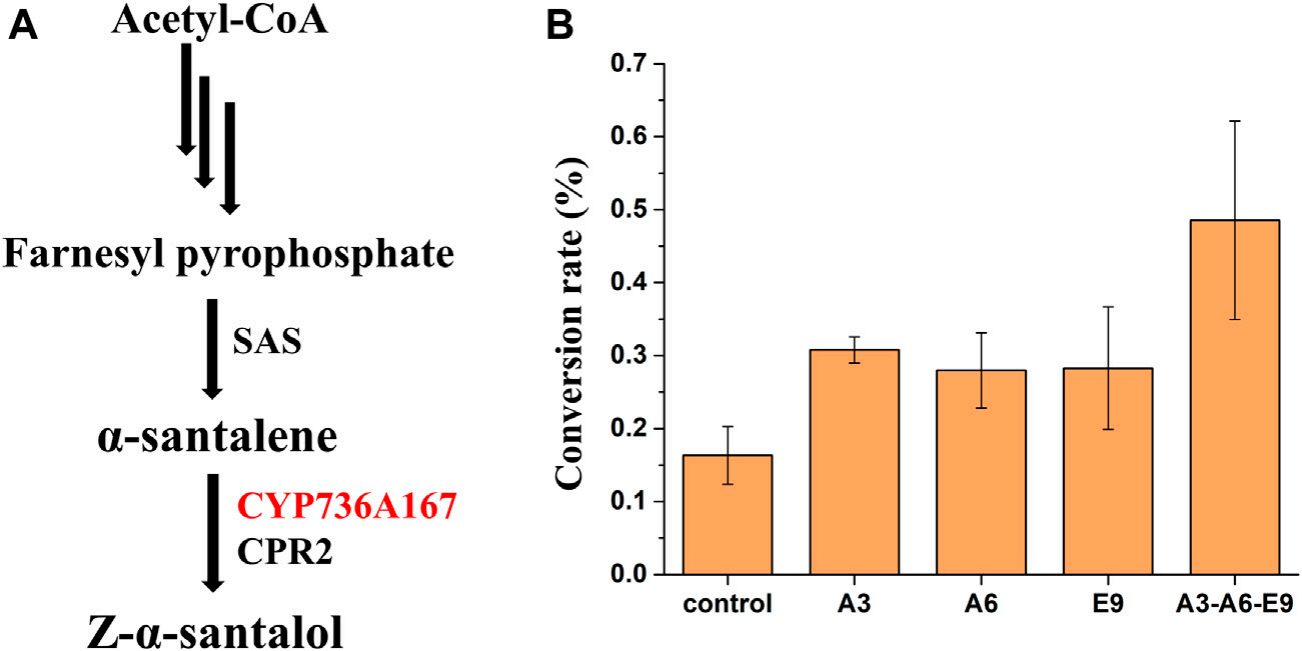
The effects of *AtGRP7* (A3), *AtMSBP1* (A6), and *AtCOL4* (E9) on the functional expression of *CYP736A167* (shown in red) and accordingly the hydroxylation of *α*-santalene to produce Z-α-santalol. **(A)** The Z-α-santalol biosynthetic pathway from acetyl-CoA. **(B)** The effects of *AtGRP7* (A3), *AtMSBP1* (A6), and *AtCOL4* (E9) on the *α*-santalene to Z-α-santalol conversion rate, either overexpressed alone (A3, A6, and E9) or in combination (A3-A6-E9). Error bars represented the mean ± s.d. of biological triplicates. SAS: *α*-santalene synthase.

To further demonstrate the general applicability of *AtGRP7*, *AtMSBP1*, and *AtCOL4* from *A. thaliana* in improving the functional expression of P450s, these genes were overexpressed in a Z-α-santalol producing yeast strain and their effects on the functional expression of *CYP736A167*, whose gene product catalyzes the hydroxylation of *α*-santalene to produce Z-α-santalol, was investigated (Figure 3A). Based on the previously constructed *α*-santalene producing strain (Dong et al., 2020), *CYP736A167* and *CPR2* were further integrated into the yeast genome to produce Z-α-santalol. To increase the accumulation of *α*-santalene, the substrate of CYP736A167 and the precursor of Z-α-santalol biosynthesis, genes involved in MVA pathway were overexpressed, including *tHMG1*, *ERG8*, *ERG10*, *ERG12*, *ERG13*, *ERG20*, *IDI1*, and *MVD1*. As shown in Supplementary Figures S3, S4, *α*-santalene was accumulated to a relatively high level, indicating that the production of Z-α-santalol was limited by the low activity of CYP736A167. The introduction of *AtGRP7* (A3), *AtMSBP1* (A6), and *AtCOL4* (E9) all demonstrated positive effects on the hydroxylation of *α*-santalene to Z-α-santalol, with the conversion rate increased for 1.89-, 1.71-, and 1.73-fold, respectively. What’s more, when these three genes (A3-A6-E9) were overexpressed simultaneously (integrated into XI2, XII2, and XI3 loci of the yeast genome), the conversion rate was the highest, representing a 2.97-fold improvement when compared with the control strain (Figure 3B). Surprisingly, the titer of Z-α-santalol was not increased as significantly as the *α*-santalene to Z-α-santalol conversion rate (Supplementary Figure S5). To figure out the possible reasons, the expression level of MVA pathway genes were profiled. As shown in Supplementary Figure S6, all the MVA pathway genes except for *ERG10* were down-regulated to different degrees. In other words, the advantage of overexpressing *AtGRP7* (A3), *AtMSBP1* (A6), and *AtCOL4* (E9) in improving the functional expression of *CYP736A167* should be combined with other metabolic engineering strategies to enhance the MVA pathway fluxes, which has been well established in yeast.

### Exploration of the Molecular Mechanisms of Improved P450 Expression in Yeast

**FIGURE 4:**
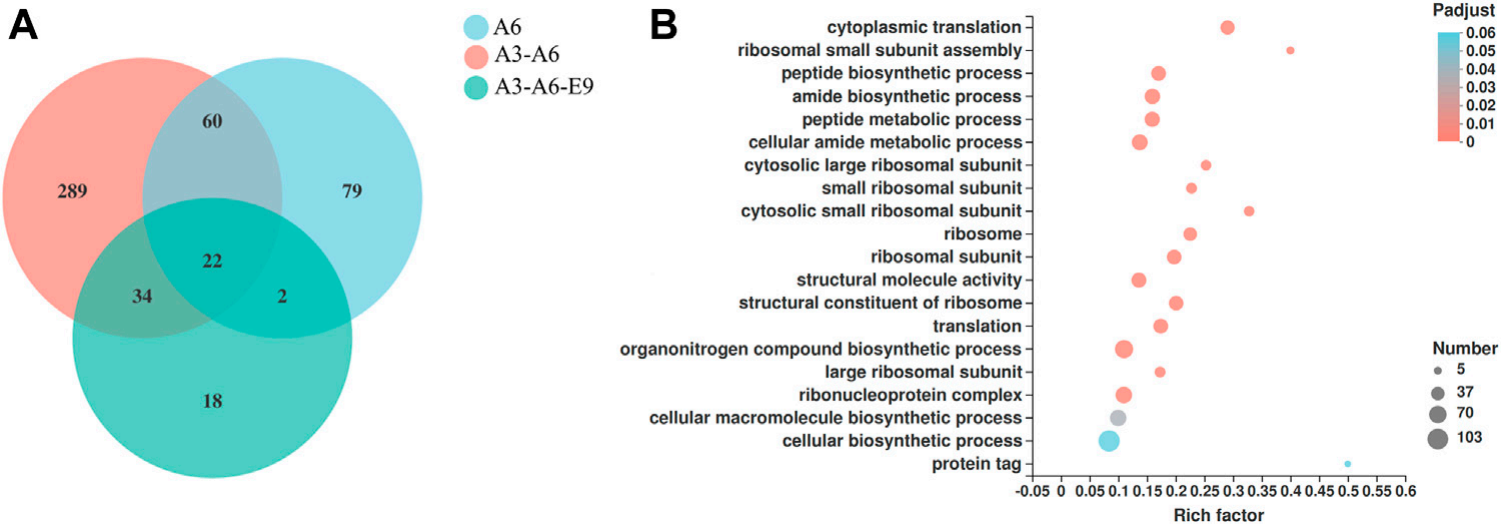
RNA-Seq analysis of the engineered strains with improved functional expression of P450s. **(A)** Venn diagram showing the number of genes with significantly different expression levels (fold change ≥2 and *p*-value < 0.05) of the engineered strains (A6, A6-A3, and A6-A3-E9) when compared with the control strain (yJS1256). **(B)** GO function enrichment bubble plot of genes that show different expression levels in yJS1256-*AtGRP7*-*AtMSBP1* (A3-A6).

Finally, the molecular mechanisms of *AtGRP7*, *AtMSBP1*, and *AtCOL4* in improving P450 expression were explored using transcriptomic analysis. mRNAs were extracted from yJS1256-*AtMSBP1* (A6), yJS1256-*AtGRP7*-*AtMSBP1* (A3-A6), and yJS1256-*AtGRP7*-*AtMSBP1*-*AtCOL4* (A3-A6-E9) and sent for next-generation sequencing (RNA-Seq). Compared with the control strain yJS1256, 163 (105 up-regulated and 58 down-regulated), 405 (317 up-regulated and 88 down-regulated), and 76 (56 up-regulated and 20 down-regulated) genes had expression level changes by more than 2-fold with a threshold of <0.05 in yJS1256-*AtMSBP1* (A6), yJS1256-*AtGRP7*-*AtMSBP1* (A3-A6), and yJS1256-*AtGRP7*-*AtMSBP1*-*AtCOL4* (A3-A6-E9) (Figure 4A and Supplementary Figure S7). Gene ontology enrichment analysis revealed that the expression level of genes associated with cytoplasmic translation, ribosomal small subunit assembly, peptide biosynthetic process, amide biosynthetic process, and peptide metabolic process were significantly changed (Figure 4B and Supplementary Figure S8). Specifically, some genes involved in integral component of membrane, such as *COX3*, *COS12*, *COB*, and *NCW1* were up-regulated. Genes related to response to stress conditions, including *GPX2* and *GRX5*, were also up-regulated. *HAC1*, a transcription factor, which was reported to induce the expression of genes related to the folding capacity of endoplasmic reticulum (ER) (Schuck et al., 2009), was apparently up-regulated. Furthermore, *MUP3* was found to be significantly up-regulated in all strains, yJS1256-*AtMSBP1* (A6), yJS1256-*AtGRP7*-*AtMSBP1* (A3-A6), and yJS1256-*AtGRP7*-*AtMSBP1*-*AtCOL4* (A3-A6-E9). *MUP3* encodes a methionine permease, whose overexpression increases methionine availability and is important to maintain the stability and abundance of membrane proteins (Lee et al., 2019). *HMX1*, an ER-localized heme oxygenase, was down-regulated in all three strains. The deletion of *HMX1* has been proved to increase heme concentration and improve the activity of CYP76AD1 and CYP102A1 in yeast (Savitskaya et al., 2019). In short, a variety of genes, including some genes with unknown functions, demonstrated significant changes at the transcriptional level, indicating complex regulatory mechanisms of the expression and folding of P450s.

## Discussion

In recent years, there is a growing interest in establishing yeast as cell factories for the production of plant natural products. Considering the significance in natural product biosynthesis as well as the low activity of P450s, various strategies have been attempted to address the challenges in functional expression of P450s in yeast. In contrast to previous strategies, such as N-terminal truncation and protein molecular engineering, the establishment of platform strains, especially those with ER expansion, has attracted more attention (Jiang et al., 2021). As most eukaryotic P450s and CPRs are membrane-bound proteins and anchored to the ER outer membranes, ER expansion has been determined to be a generally applicable strategy to improve the functional expression of P450s Emmerstorfer et al. found that the overexpression of *ICE2* could increase the stability and activity of P450/CPR (Emmerstorfer et al., 2015). DNA repair and recombination gene *RAD52* was also reported to improve the functional expression level of P450s (Wriessnegger et al., 2016). The overexpression of *IN O 2* and the deletion of *PAH1* and *OPI1*, which had been found to enlarge the ER, could also improve the functional expression of P450s (Schuck et al., 2009; Arendt et al., 2017; Kim et al., 2019). Therefore, the identification of novel genetic engineering targets is a promising strategy for the construction of platform strains for functional expression of s wide variety of P450s.

Considering the complex regulatory machinery in P450 expression and folding, genome-scale engineering has been proved as an effective strategy to overcome our limited knowledge and identify new engineering targets (Lian et al., 2018; Lian et al., 2019). Among various genome-scale engineering strategies, cDNA overexpression library is the simplest and most commonly employed, with the advantages of high-level expression and the introduction of heterologous genes to enable improved and even novel phenotypes of interests. Baumann et al. successfully screened two new targets that could increase the titer of octanoic acid by overexpressing the yeast cDNA library in the octanoic acid-producing *S. cerevisiae* (Baumann et al., 2021). Similarly, Shi et al. overexpressed the *Yarrowia lipolytica* cDNA library and identified key targets that could improve the production of fatty acids in *S. cerevisiae* (Shi et al., 2016). Encouraged by these successful examples, the present study screened the *A. thaliana* cDNA library to identify the key genes that were closely related to functional expression of P450s. Using the biosensor-based high throughput screening method, three target genes from *A. thaliana* (*AtGRP7*, *AtMSBP1*, and *AtCOL4*) were identified and proved to be effective in improving the functional expression of *CYP76AD1* and *CYP736A167*, whose gene products had been determined to be rate-limiting for the production of betaxanthin and Z-α-santalol, respectively. The positive role of these genes in improving the functional expression of P450s with different origins (*CYP76AD1* from *B. vulgaris* and *CYP736A167* from *S. album*) indicated the potential of general applicability in functional expression of a wide variety of P450s.

In conclusion, an *A. thaliana* cDNA library was successfully expressed in yeast, and three target genes *AtGRP7*, *AtMSBP1*, and *AtCOL4* from *A. thaliana* were identified to be effective in improving the functional expression of CYP76AD1 and accordingly the production of betaxanthin. More importantly, these target genes could also effectively increase the activity of CYP736A167, catalyzing the hydroxylation of *α*-santalene to produce Z-α-santalol, whose conversion rate was increased for 2.97-fold when these three genes were overexpressed simultaneously. The target genes identified in the present study promise the construction of a platform yeast strain for functional expression of P450s and accordingly production of natural products.

## Data Availability

The raw reads of the NGS data were deposited into the NCBI Sequence Read Archive (SRA) database (accession number: PRJNA760804).

## Supplementary Material

Supplementary Table S1 List of plasmids used in the study.

Supplementary Table S2 Primers used in this study.
