# Supplementary material for: Improved Functional Expression of Cytochrome P450s in Saccharomyces cerevisiae Through Screening a cDNA Library From Arabidopsis thaliana
Source: Front Bioeng Biotechnol. 2021 Dec 9;9:764851. doi: 10.3389/fbioe.2021.764851 (PMC8696027; doi:10.3389/fbioe.2021.764851)
Supplement: Supplementary file 1 [file DataSheet1.PDF]

SUPPLEMENTARY INFORMATION

A B

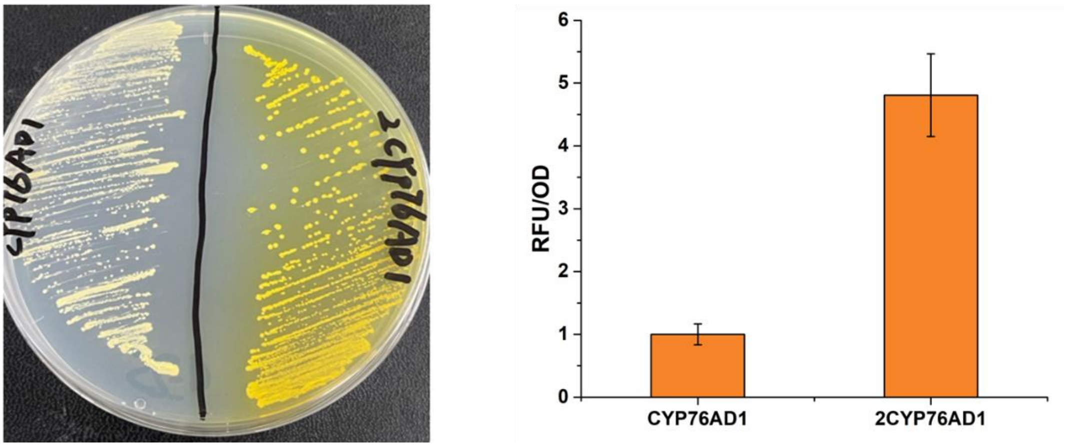

Supplementary Figure S1. Verification of CYP76AD1 as the rate-limiting enzyme for betaxanthin biosynthesis in yeast. An additional copy of *CYP76AD1*$^{W13L\text{-}F309L}$ expression cassette was integrated into the chromosome of the biosensor strain (yJS1256). The production of betaxanthin was compared by the yellow color densities (A) and fluorescence intensities (B). With the introduction of an additional copy of CYP76AD1, the production of betaxanthin was significantly improved, indicating that CYP76AD1 was rate-limiting for betaxanthin biosynthesis in yeast. Similarly, if the overexpression of a plant gene can improve the functional expression of *CYP76AD1*, the production of betaxanthin should be increased as well. In other words, yJS1256 can be used as the biosensor strain for high throughput screening of mutant yeast strains with improved functional expression of P450s.

A

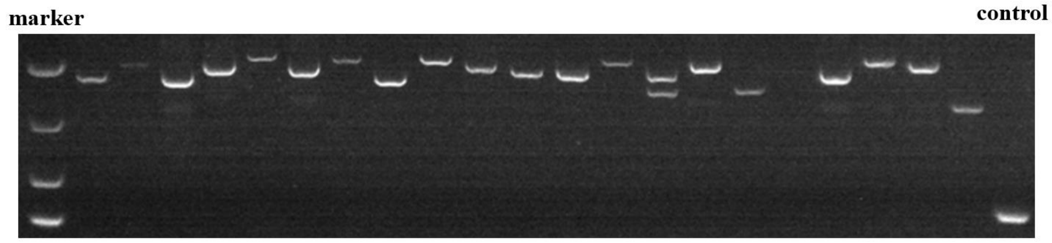

B

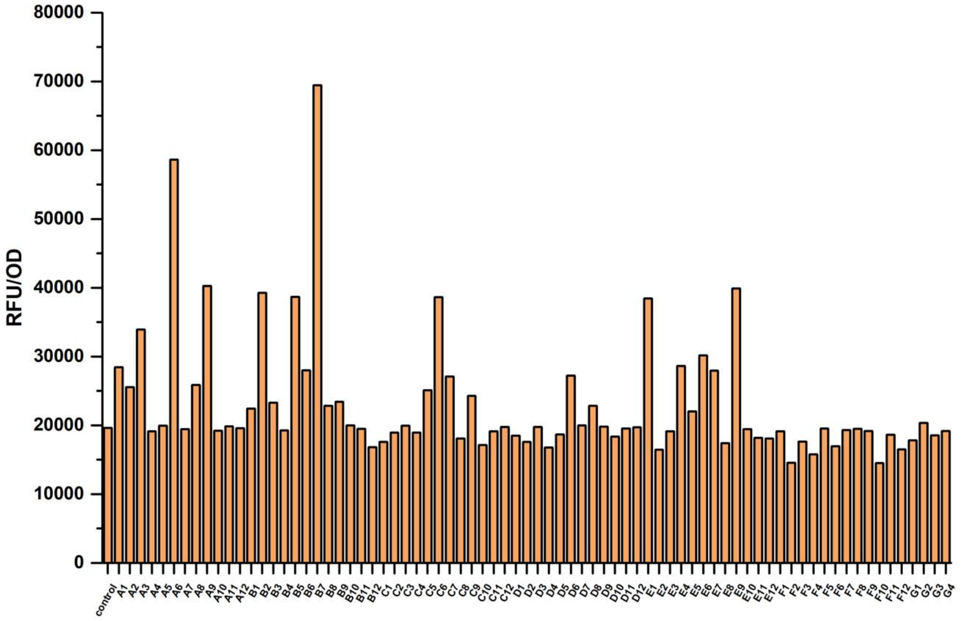

Supplementary Figure S2. High throughput screening based on the betaxanthin-producing yeast (biosensor strain) to obtain *A. thaliana* genes that can improve the expression of P450s. **(A)** PCR verification of the diversity of *A. thaliana* cDNA library transformed into *S. cerevisiae*. **(B)** Fluorescence intensities of 76 clones with the highest yellow color intensities selected from agar plates. RFU/OD refers to the fluorescence intensity normalized to cell density.

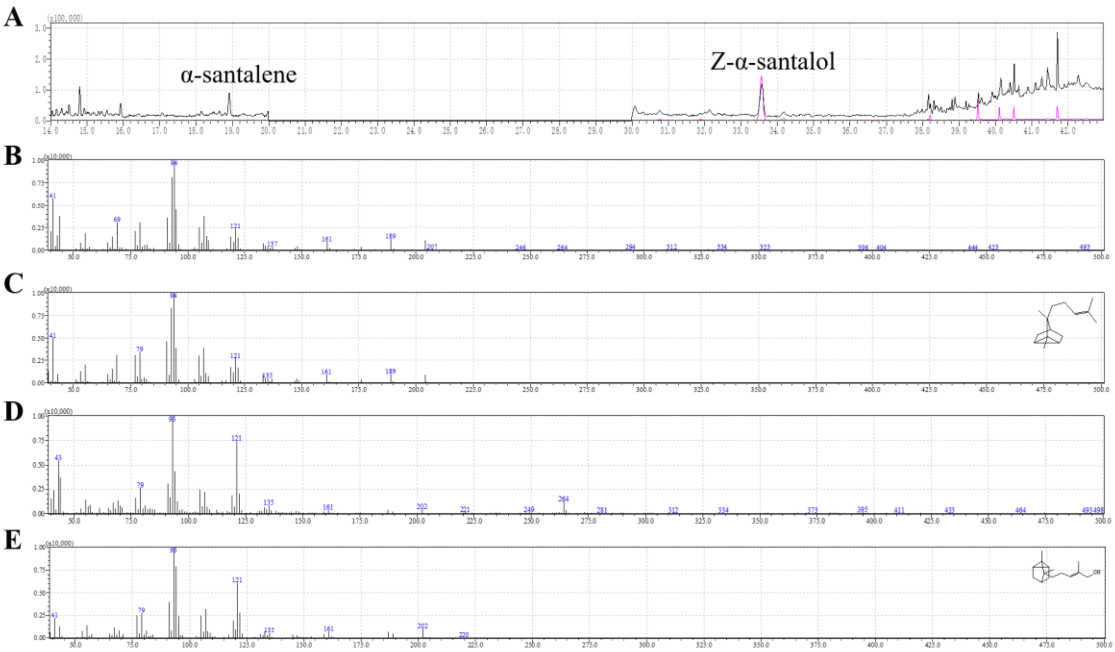

Supplementary Figure S3. Quantification of the production of α-santalene and Z-α-santalol in yeast. **(A)** GC-MS spectrogram of α-santalene and Z-α-santalol. **(B)** Mass spectra of α-santalene in the fermentation sample. **(C)** Mass spectra of α-santalene standard. **(D)** Mass spectra of Z-α-santalol in the fermentation sample. **(E)** Mass spectra of Z-α-santalol standard.

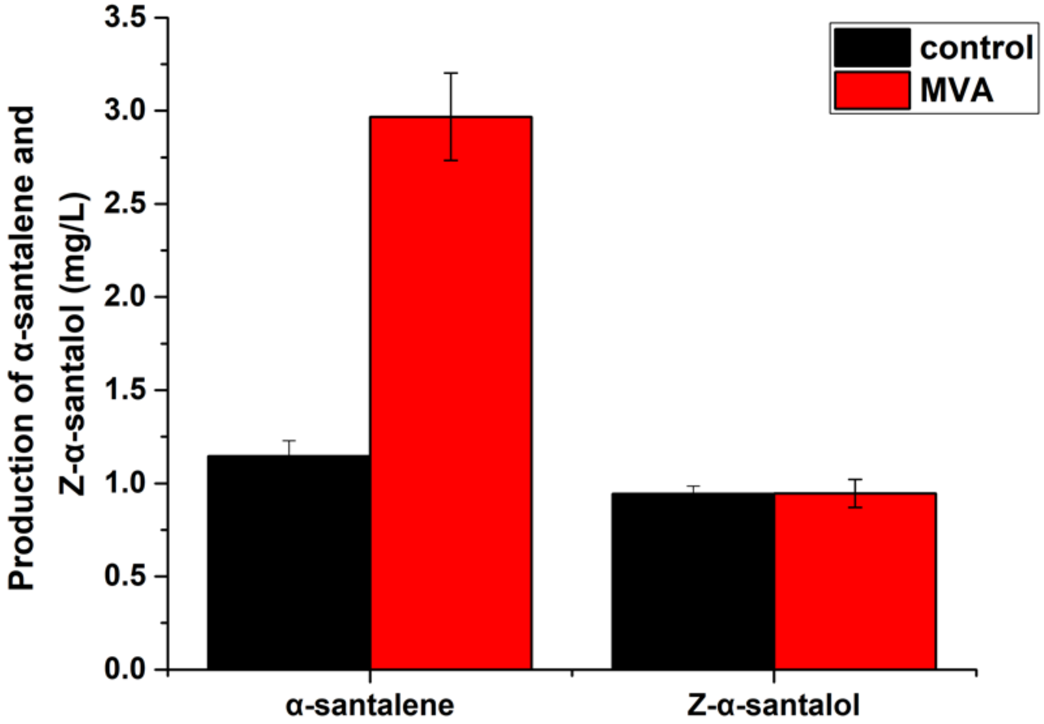

Supplementary Figure S4. Verification of CYP736A167 as the rate-limiting enzyme for Z-α-santalol biosynthesis in yeast. The production of α-santalene and Z-α-santalol in yeast strains with (MVA strain) or without (Control strain) the overexpression MVA pathway genes was compared. The introduction of MVA pathway genes significantly increased the accumulation of α-santalene, while the production of Z-α-santalol was not increased, indicating that the production of Z-α-santalol in yeast was limited by the hydroxylation of α-santalene by CYP736A167.

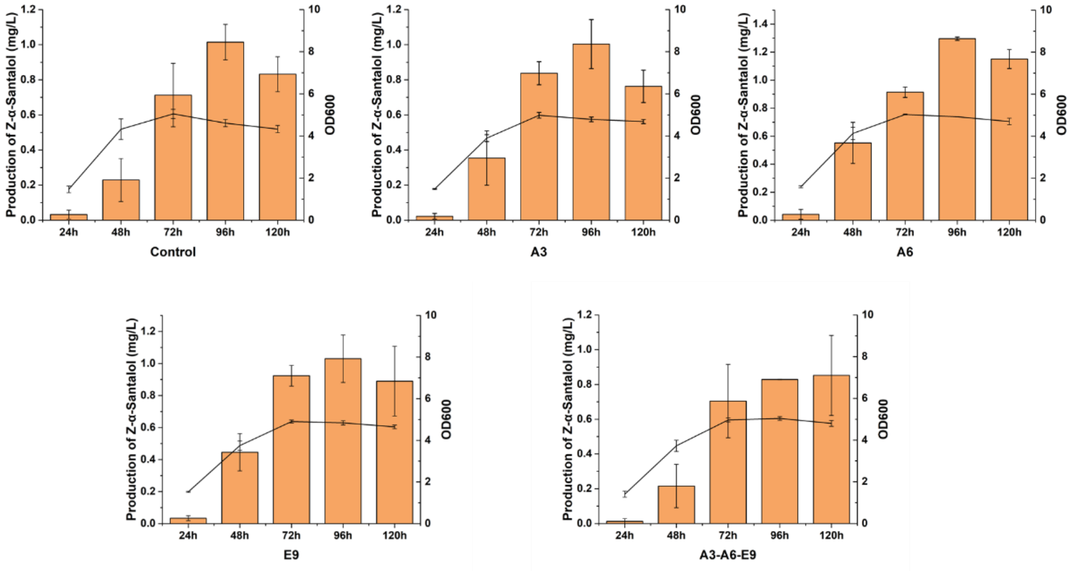

Supplementary Figure S5. The cell growth (curve) and production of Z-α-santalol (column) in the yeast strain with *AtGRP7* (A3), *AtMSBP1* (A6), and *AtCOL4* (E9) expressed individually or simultaneously.

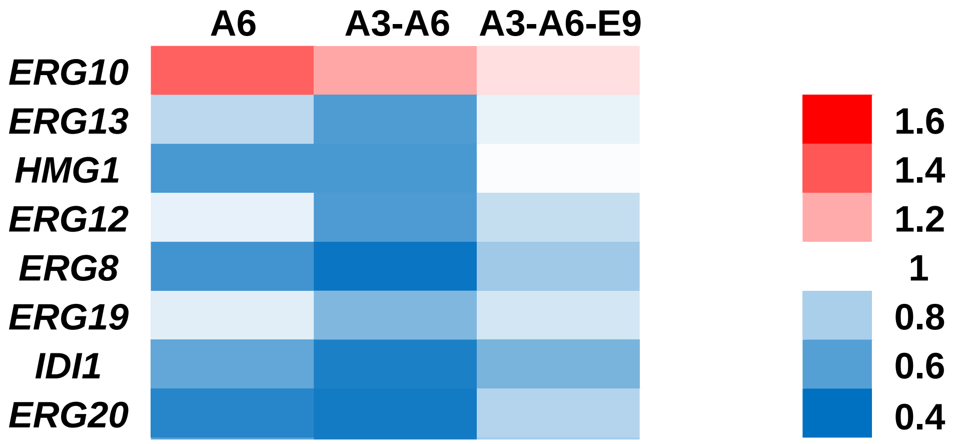

Supplementary Figure S6. Profiling of the expression level of MVA pathway in the engineered strains, A6, A3-A6, and A3-A6-E9. Based on the RNA-Seq results, the expression level of MVA pathway genes (*ERG10, ERG13, HMG1, ERG12, ERG8, ERG19, IDI1*, and *ERG20*) was compared among the control strain and engineered strains (A6, A3-A6, and A3-A6-E9).

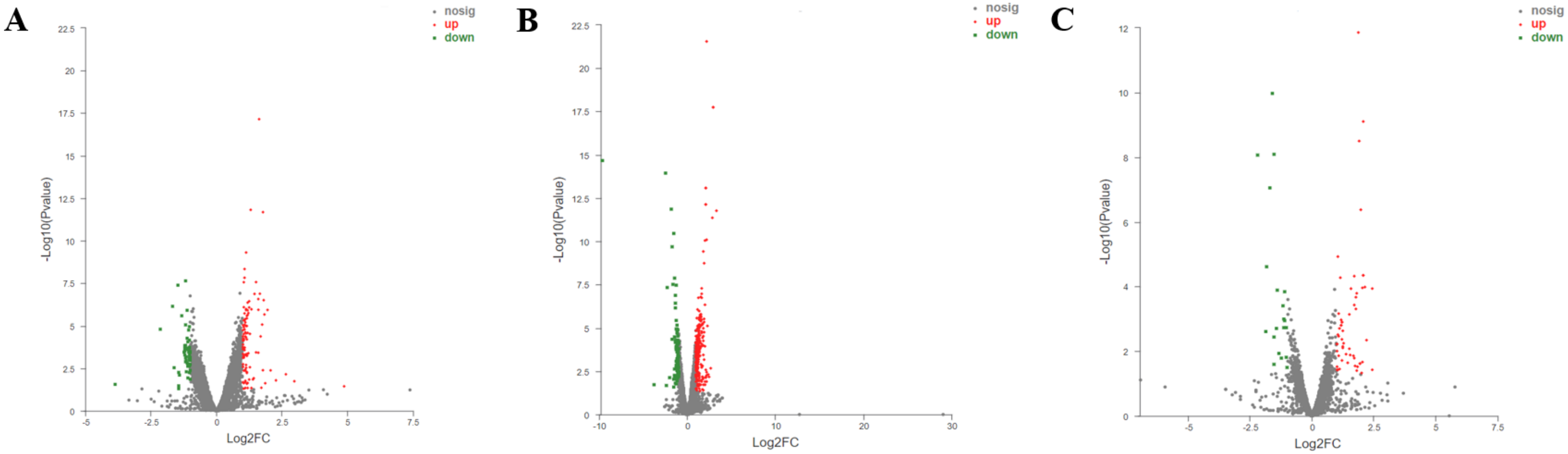

Supplementary Figure S7. Volcano maps of genes that show different expression levels in **(A)** yJS1256-*AtGRP7* (A3), **(B)** yJS1256-*AtGRP7*-*AtMSBP1* (A3-A6), and **(C)** yJS1256-*AtGRP7-AtMSBP1-AtCOL4* (A3-A6-E9).

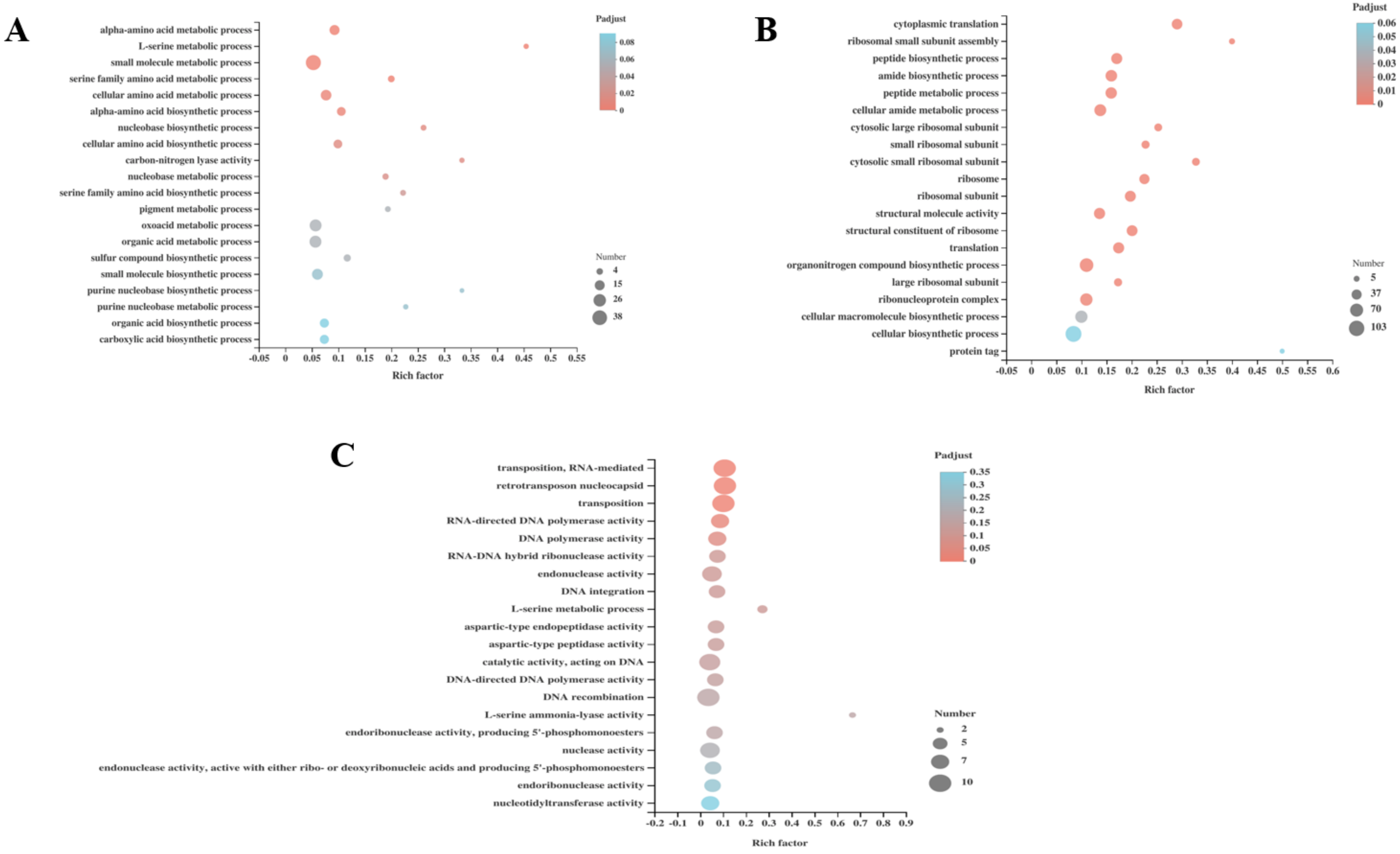

Supplementary Figure S8. GO function enrichment bubble plots of genes that show different expression levels in **(A)** yJS1256-*AtGRP7* (A3), **(B)** yJS1256-*AtGRP7-AtMSBP1* (A3-A6), and **(C)** yJS1256-*AtGRP7-AtMSBP1-AtCOL4* (A3-A6-E9).

Supplementary Table S1. List of plasmids used in the study.

Name	Description	Reference
pRS415	CEN/ARS, *LEU2*	This lab
pRS416	CEN/ARS, *URA3*	This lab
pRS416-TEF1p	Helper plasmid for *A. thaliana* cDNA library expression	This study
pGADT7-AD	*A. thaliana* cDNA library	
pRS415-CYP736A167	*TPI1p-CYP736A167-TPI1t*	This study
pRS415-CPR2	*TEF1p-CPR2-TEF1t*	This study
pRS413-Cyto6	*GPM1p-tHMG1-ADH1t*<br>*GPDp-ERG8-CYC1t*<br>*ENO2p-ERG13-PGK1t*<br>*TPI1p-ERG20-TPI1t*<br>*TEF1p-ERG12-TEF1t*	(Dong et al. 2021)
pRS413-Cyto5	*GPM1p-EGR10-ADH1t*<br>*GPDp-MVD1-CYC1t*<br>*ENO2p-IDI1-PGK1t*<br>*TPI1p-tHMG1-TPI1t*	(Dong et al. 2021)
pRS416-A3	*TEF1p-AtGRP7-ADH1t*	This study
pRS416-A6	*TEF1p-MSBP1-ADH1t*	This study
pRS416-E9	*TEF1p-AtCOL4-ADH1t*	This study
pRS426-SaSg336	gRNA for X4 integration site	(Lian et al. 2019)
pRS426-SaSg414	gRNA for XI2 integration site	(Lian et al. 2019)
pRS423-SaSg339	gRNA for XI3 integration site	(Lian et al. 2019)
pRS426-SaSg341	gRNA for XII2 integration site	(Lian et al. 2019)
pRS426-SaSg416	gRNA for XII4 integration site	(Lian et al. 2019)
pRS425-SaSg343	gRNA for XII5 integration site	(Lian et al. 2019)

Supplementary Table S2 Primers used in this study.

Name	Sequence (5'-3')
TEF1p-F	NNNNNGGTACCATAGCTTCAAAATGTTTCTACTC
TEF1p-R	NNNNNGAGCTCCATAGGGTAGGGGAATTTCGACCGGGATCCCGTATCCATGGAATTCATATGAGCGTAATCTGGTACGTCGTATGGGTATTTGTAATTAAAACTTAGATTAGATTGC
cDNA-F	TACCCATACGACGTACCAGATTAC
cDNA-R	GAGCTCCATAGGGTAGGGGAATTTCG
CYP736A167-XII5-F	TTTCTAACTCTTCTCACGCTGCCCCTATCTGTTCTTCCGCTATATCTAGGAACCCATCAGGTTG
CYP736A167-R	GATTGCTATGCTTTCTTTCTAATGAGCAAGAAG
CPR2-F	CCGCGGATAGCTTCAAAATGTTTCTACTC
CPR2-XII5-R	CTAGCCTTATTGTTTTAGTTCAGTGACAGCGAACTGCCGTATAGCGCCGATCAAAGTAT
Cyto5-X4-F	CAGCCACAGTTGTAGTCACGTGCGCGCCATGCTGACTAATTAGTCGTGCAATGTATGAC
Cyto5-homo-R	TCCTTCCTTTTCGGTTAGAG
Cyto5-homo-F	AGGAAACAATGATGGATTCC
Cyto5-X4-R	TGGTAGTTGGAGCGCAATTAGCGTATCCTGTACCATACTACCGCGGTATATAACAGTTG
Cyto6-XI3-F	CCAATCAAAGAAGCATCGGTTCAGATCGAGCAAACTGTAGTAGTCGTGCAATGTATGAC
Cyto6-homo-R	GTTGCTTTTGCGGCCTAAGT
Cyto6-homo-F	GTATTTATAGCAAACGCAATTG
Cyto6-XI3-R	TGACATCCAAACTACAAAACCGAGATTGGACATATAGCACGATAGCGCCGATCAAAGTA
A3-XII5-F	TTTCTAACTCTTCTCACGCTGCCCCTATCTGTTCTTCCGCGATAGCTTCAAAATGTTTC
A3-XII5-R	CTAGCCTTATTGTTTTAGTTCAGTGACAGCGAACTGCCGTCCGGTAGAGGTGTGGTCAA
A6-X4-F	CAGCCACAGTTGTAGTCACGTGCGCGCCATGCTGACTAATGATAGCTTCAAAATGTTTC
A6-X4-R	TGGTAGTTGGAGCGCAATTAGCGTATCCTGTACCATACTACCGGTAGAGGTGTGGTCAA
E9-XI3-F	CCAATCAAAGAAGCATCGGTTCAGATCGAGCAAACTGTAGGATAGCTTCAAAATGTTTC
E9-XI3-R	TGACATCCAAACTACAAAACCGAGATTGGACATATAGCACCCGGTAGAGGTGTGGTCAA
A6-XII2-F	TGCGTCTAACGCTTTTGCCACTTGGATTTCTATTATAGGAATAGCTTCAAAATGTTTCT
A6-XII2-R	AAGAAATTCTTCCTGTGCTTCATCAAAACGCGAAAATTCGCCGGTAGAGGTGTGGTCAA

E9-XII4-F	ACTTTGTACTATTCCTTCCCCGTTTACTCAATTCTTGAAGATAGC TTCAAAATGTTTCT
E9-XII4-R	AGCGCGGGTAACATACCTCCGTGAGGCATCCTTTTATTTGCCGGT AGAGGTGTGGTCAA

Supplementary References

Dong, C., Shi, Z., Huang, L., Zhao, H., Xu, Z., Lian, J. (2021). Cloning and characterization of a panel of mitochondrial targeting sequences for compartmentalization engineering in *Saccharomyces cerevisiae*. *Biotechnol. Bioeng*. doi:10.1002/bit.27896

Lian, J., Schultz, C., Cao, M., HamediRad, M., Zhao, H. (2019). Multi-functional genome-wide CRISPR system for high throughput genotype-phenotype mapping. *Nat. Commun*. 10, 5794. doi:10.1038/s41467-019-13621-4
